# Molecular mechanisms regulating T-cell receptor signaling and T-cell function

**DOI:** 10.3389/fimmu.2026.2002535

**Published:** 2026-10-27

**Authors:** Vasily V. Kurilin, Marina S. Fisher, Sergey V. Sennikov

**Affiliations:** 1 Laboratory of Molecular Immunology, Federal State Budgetary Scientific Institution “Research Institute of Fundamental and Clinical Immunology”, Novosibirsk, Russia; 2 Laboratory of Immune Engineering, Institute of Medicine and Medical Technologies, Novosibirsk State University, Novosibirsk, Russia

**Keywords:** functional avidity, immunometabolism, pMHC density, signal transduction, T cell receptor, T-cell exhaustion, TCR signaling, TCR-T cells

## Abstract

The T-cell receptor (TCR) is the central mediator of antigen-specific recognition and a key regulator of T-lymphocyte function. Current evidence indicates that TCR signaling is not a linear cascade but a multilevel dynamic system in which cellular responses are shaped by the combined effects of TCR-CD3 structural organization, spatial receptor distribution, the lipid microenvironment, mechanical forces, kinase and phosphatase activity, co-stimulatory and inhibitory receptors, cellular metabolic state, and the strength and duration of TCR interactions with peptide-MHC (pMHC) complexes. This review summarizes current concepts of the molecular and spatial organization of TCR-CD3; mechanisms of signal initiation involving Lck, CD45, SHP phosphatases, ITAMs, ZAP-70, and the LAT-SLP-76 signalosome; and the coupling of TCR signaling to T-cell metabolic programs. Particular attention is paid to the nonlinear relationship between functional responses, TCR affinity, and target pMHC density; the role of chronic antigenic stimulation in T-cell exhaustion and activation-induced cell death (AICD); and the involvement of NFAT-, TOX-, NR4A-, and TCF1-dependent programs in this process. We also discuss the translational relevance of these mechanisms to antitumor immunotherapy and the restoration of immune tolerance, including immune checkpoint blockade, CAR-T, TCR-T, and TCR-Treg approaches and CD3/TCR-directed T-cell-engager platforms. Finally, we consider the concept of TCR signal tuning, according to which therapeutic efficacy is determined not by maximal receptor signal strength but by an optimal balance between antigen sensitivity, specificity, effector activity, metabolic fitness, and the ability of T cells to retain function under sustained antigenic pressure.

## 1 Introduction

The vertebrate adaptive immune system has a unique capacity to recognize an almost unlimited diversity of antigens and establish long-term immunological memory. T lymphocytes play a central role in these processes, and their functional activity is controlled by the T-cell receptor (TCR). The TCR complex is a multisubunit membrane structure that not only mediates specific recognition of antigenic peptides presented by major histocompatibility complex molecules (pMHC) but also initiates intracellular signaling cascades that determine T-cell fate (1, 2).

Accordingly, this review aims to integrate structural, spatial, biochemical, metabolic, and temporal determinants of TCR signaling into a common framework for understanding T-cell functional fate. Rather than considering receptor triggering, metabolic reprogramming, and exhaustion as separate processes, we examine how TCR-pMHC affinity and kinetics, cognate pMHC density, kinase-phosphatase balance, co-stimulatory and inhibitory inputs, and cellular metabolism jointly shape signal strength and duration. Particular emphasis is placed on the implications of this integrated view for TCR-engineered T cells, including the definition of a functional signaling window that supports antigen sensitivity and effector activity while limiting chronic overstimulation and loss of persistence.

In contrast to reviews in which TCR structural organization, proximal signaling, metabolic adaptation, and T-cell functional outcomes are considered largely as separate regulatory layers, the present work analyzes these processes within a unified framework of TCR signal tuning. Particular attention is given to how the molecular and spatial organization of TCR-CD3 and the signalosome determines signal amplitude, duration, and quality, and how these parameters are related to TCR affinity, pMHC density, and cellular functional state. This approach links fundamental mechanisms of TCR signaling to the rational tuning of TCR-T cells, CAR-T cells, and other T-cell therapeutic platforms.

## 2 Structural organization of the TCR complex

Within the TCR complex, antigen recognition and signal transduction are partitioned among distinct subunits: the variable domains of TCRα and TCRβ bind ligand, whereas the CD3γϵ, CD3δϵ, and CD3ζζ dimers mediate intracellular signal transmission. Complex assembly is maintained by specific polar interactions between the transmembrane domains of TCRαβ and CD3 (2, 3).

The TCRαβ heterodimer has short cytoplasmic tails that lack intrinsic signaling capacity. In contrast, the CD3 subunits contain ITAMs: one each in CD3ϵ, CD3γ, and CD3δ, and three in each CD3ζ chain, yielding a total of ten ITAMs per TCR complex and enabling signal amplification (2, 3).

### 2.1 Molecular mechanisms of TCR signaling

The mechanism linking pMHC engagement of the TCR to phosphorylation of CD3 ITAMs remains debated. In addition to kinetic proofreading and receptor clustering, increasing importance is attributed to ligand-induced allosteric rearrangements within TCR-CD3 (4, 5).

An important additional feature is TCR mechanosensitivity: forces generated during contact between a T cell and an antigen-presenting cell can stabilize specific receptor conformations and increase the accessibility of functionally relevant CD3 motifs (6, 7).

A key event in early signaling is recruitment of Lck. In addition to its association with coreceptors, CD3ϵ contains an RK motif implicated in Lck interaction and, upon activation, exposes a proline-rich region that binds Nck (4, 8, 9).

### 2.2 Heterogeneity of TCR-expressing populations

In addition to the dominant αβ TCR, γδ T cells express γδ TCRs and often recognize antigens independently of classical MHC molecules. Although both receptor types use common CD3 signaling components, the distinctive features of proximal γδ TCR signaling remain less well characterized (10). γδ T cells are considered here primarily to illustrate that core CD3-dependent signaling principles are shared across TCR lineages despite substantial differences in ligand recognition. Comparative analysis of αβ and γδ TCRs may therefore help distinguish broadly conserved mechanisms of receptor triggering from context-specific features. Because mechanistic evidence is considerably more extensive for αβ T cells, the subsequent sections focus predominantly on αβ TCR signaling.

TCR signaling mechanisms have direct clinical relevance: their disruption is associated with immunodeficiency and autoimmune pathology, while principles of CD3 signaling architecture are exploited in the design of CAR-T cells (2, 11).

## 3 Spatial organization of the TCR complex

The T-cell receptor (TCR) is a highly organized multisubunit membrane complex in which antigen recognition and signal transduction are spatially segregated among distinct polypeptide chains. In αβ T cells, the antigen-recognition module is formed by the TCRαβ heterodimer, whereas signaling is mediated by the associated CD3γϵ, CD3δϵ, and CD3ζζ dimers. Thus, the functional TCR-CD3 complex is a heterooctamer with a TCRαβ:CD3γϵ:CD3δϵ:CD3ζζ stoichiometry of 1:1:1:1 (12, 13). Determination of the intact human TCR-CD3 structure by cryo-electron microscopy (cryo-EM) at 3.7 Å resolution was a major advance in understanding receptor organization and demonstrated that its extracellular and transmembrane regions form a single structurally integrated complex (12).

### 3.1 Multilevel architecture of the TCR-CD3 complex

Spatially, TCR-CD3 can be viewed as a structure comprising three functionally connected levels. The most membrane-distal level is formed by the Vα and Vβ variable domains, which contain complementarity-determining regions (CDRs) and directly engage peptide-MHC (pMHC) complexes. Classical crystallographic studies of TCR-pMHC complexes revealed a predominantly diagonal orientation of the TCR over the peptide-binding groove of MHC (14). This orientation is retained in the complete membrane complex, although pMHC and TCR are positioned at a pronounced angle relative to the surfaces of the interacting cells, underscoring the importance of intermembrane distance and the spatial geometry of the immunological contact (15, 16).

The intermediate, membrane-proximal extracellular level is formed by the TCR Cα and Cβ constant domains together with the immunoglobulin-like domains of CD3γϵ and CD3δϵ. The latter are positioned asymmetrically on either side of TCRαβ and interact primarily with the constant domains and connecting regions of the TCR. Cryo-EM analysis showed that TCRαβ, CD3γϵ, and CD3δϵ form a compact membrane-proximal structure that substantially restricts independent movement of the individual subunits (12). Additional stabilization is provided by membrane-proximal connecting segments and disulfide bonds. In the complete pMHC-TCR-CD3 structure, these interactions form an interconnected network of contacts that mechanically links the antigen-recognition module to the transmembrane region (15).

The third structural level consists of eight transmembrane α-helices. The transmembrane region is the principal assembly hub of the entire TCR-CD3 complex. CD3γϵ, CD3δϵ, and CD3ζζ form a ring-like or barrel-shaped arrangement around the transmembrane segments of TCRαβ (12). Its stability is maintained by a combination of hydrophobic interactions and highly conserved electrostatic contacts. Positively charged residues in the TCRα and TCRβ transmembrane segments interact with pairs of negatively charged residues in the CD3 subunits. The requirement for these interactions was demonstrated before the complete receptor structure became available: disruption of charged transmembrane motifs impaired the sequential incorporation of CD3δϵ, CD3γϵ, and CD3ζζ into the mature receptor complex (13). Subsequent cryo-EM data directly confirmed this model (12).

### 3.2 The lipid microenvironment as a structural component of the TCR

A major extension of the classical protein-centric view of the TCR came from the discovery of specific receptor interactions with plasma membrane lipids. In the cryo-EM structure of the complete TCR-pMHC complex, electron density consistent with a sterol lipid was observed between the transmembrane helices (15). More detailed structural studies identified a cholesterol-binding site within the transmembrane core of TCR-CD3 that can restrict CD3ζ mobility (17). According to the proposed model, cholesterol stabilizes one of the inactive conformations of the complex and thereby reduces the probability of CD3ζ ITAM phosphorylation. Mutations that disrupt this interaction increased mobility of the ζ-chain transmembrane segment and enhanced basal receptor activation (17). These findings are consistent with earlier biochemical data showing that cholesterol binding to TCRβ can shift the receptor toward an inactive state (18).

Particularly informative in this context are cryo-EM data reported in 2025 for TCR-CD3 reconstituted in lipid nanodiscs (19). Unlike previously studied detergent-solubilized complexes, the unliganded TCR in a membrane-like lipid environment predominantly adopted two closely related closed and compact conformations. Compared with the open state, its extracellular region was approximately 35 Å shorter along the axis perpendicular to the membrane. Binding of an HLA-peptide complex shifted the TCR into a more open and extended conformation. Moreover, artificial locking of the closed configuration by disulfide bonds reduced maximal ligand-dependent T-cell activation (19). These findings provide some of the most direct structural evidence to date for allosteric rearrangement of the complete TCR-CD3 complex upon binding a physiological ligand.

Notably, this observation partially differs from earlier cryo-EM studies of detergent-solubilized TCR-pMHC complexes, in which no major global rearrangements were detected between the free and bound states (15, 16). The discrepancy may therefore reflect not merely methodological differences but an important role of the lipid bilayer in shaping the TCR energy landscape. The most plausible current view is not that of two absolutely fixed “off” and “on” structures, but rather of a dynamic conformational ensemble whose relative populations depend on pMHC engagement, the lipid environment, and the mechanical state of the receptor.

Glycosylation may provide an additional level of spatial stabilization of the TCR. Multiple N-linked glycans have been identified on TCRα, TCRβ, and CD3 subunits in structures of the complete receptor (16). Nanodisc structures reported in 2025 revealed conformation-dependent glycan-glycan contacts, suggesting that carbohydrate chains may help stabilize particular receptor states (19). Thus, TCR glycosylation should be considered not only in the context of receptor biosynthesis and trafficking but also potentially as an element of its spatial organization.

### 3.3 Spatial positioning of CD3 cytoplasmic domains

The cytoplasmic domains of CD3γ, CD3δ, two CD3ϵ chains, and two CD3ζ chains contain a total of ten immunoreceptor tyrosine-based activation motifs (ITAMs), which form the molecular platform for signal initiation. These CD3 cytoplasmic regions are largely intrinsically disordered and, because of their high conformational mobility, are poorly resolved in cryo-EM structures of the complete TCR-CD3 complex (12, 15, 16). Their spatial organization is determined by interactions both among protein components of the complex and with the inner leaflet of the plasma membrane.

For CD3ϵ, clusters of basic residues mediate electrostatic association of the cytoplasmic domain with negatively charged membrane lipids. NMR analysis showed that, in the membrane-bound state, tyrosine residues within the CD3ϵ ITAM can become partially embedded in the hydrophobic region of the lipid bilayer, thereby limiting their accessibility to Src-family kinases (20). Membrane binding has also been demonstrated for the CD3ζ cytoplasmic domains (21). Thus, membrane-dependent spatial shielding of ITAMs may represent one mechanism that prevents spontaneous signal initiation in resting T cells.

TCR stimulation induces local changes in the lipid environment of receptor microclusters, including reduced availability of phosphatidylserine and decreased negative charge of the inner membrane leaflet. This is accompanied by dissociation of the CD3ϵ cytoplasmic domain from the membrane and increased accessibility of its ITAM (21). The transition of cytoplasmic domains from membrane-bound to more exposed states should be viewed as a dynamic equilibrium rather than irreversible simultaneous release of all ITAMs. Such a model is consistent with the high sensitivity of the TCR to subtle changes in local membrane organization.

Additional indirect evidence for structural rearrangement of the receptor cytoplasmic region is the ligand-induced exposure of the proline-rich sequence in CD3ϵ that mediates binding of the adaptor protein Nck (9). Although the universal functional requirement for this interaction across all forms of TCR signaling remains debated, the change in accessibility of the CD3ϵ region itself provides further evidence for dynamic reorganization of cytoplasmic elements of the complex during activation.

### 3.4 Nanoscale organization of the TCR in the plasma membrane

Spatial organization of the TCR is not limited to the structure of an individual octameric complex. Super-resolution microscopy has shown that receptors are distributed non-uniformly across the T-cell surface. In resting cells, a fraction of TCRs localizes to small membrane domains or “protein islands” that are spatially segregated from some downstream signaling components, including LAT (22). Upon activation, these domains move together and concatenate to form signaling microclusters.

The functional significance of receptor compaction was demonstrated by single-molecule localization microscopy. The probability of TCR-CD3 phosphorylation increased as the distance between neighboring receptor complexes decreased, and the densest nanoclusters displayed the highest signaling activity (23). Cluster density depended on both pMHC concentration and the properties of TCR-pMHC interactions. Spatial receptor reorganization may therefore provide an additional level of antigen discrimination.

However, the extent of pre-existing TCR oligomerization in resting cells remains controversial and depends on the imaging and analytical methods used. It is therefore more appropriate to describe a dynamic nanoscale heterogeneity of TCR distribution, encompassing both individual complexes and areas of locally increased receptor density, rather than a single fixed oligomeric state.

A substantial fraction of TCRs is localized to T-cell microvilli (24). Microvilli function as dynamic sensory structures that rapidly scan the antigen-presenting cell surface; detection of cognate pMHC stabilizes the corresponding area of cell-cell contact and promotes local TCR accumulation (25). Thus, T-cell surface topology provides pre-existing spatial enrichment of receptors at sites that are most actively involved in antigen search.

Following productive antigen recognition, TCRs form larger signaling microclusters containing phosphorylated CD3, ZAP-70, LAT, and other components. Super-resolution microscopy has shown that these microclusters are themselves heterogeneous: individual signaling proteins occupy spatially distinct nanodomains, and their assembly proceeds sequentially (26). Thus, even after TCRs coalesce into microclusters, an internal spatial organization persists that can regulate signaling kinetics and intensity.

### 3.5 Mechanical component of TCR spatial organization

The architecture of TCR-CD3 enables the receptor to sense not only the chemical properties of pMHC binding but also mechanical forces generated at the immunological synapse. Experimental application of force directly to the TCR can initiate Src-dependent signaling (27). Moreover, agonist TCR-pMHC pairs can form so-called catch bonds, whose lifetimes increase within a defined force range, whereas weaker ligands more often show the opposite behavior (28). This provides an additional mechanism for amplifying differences between agonist and non-agonist pMHC ligands.

Structurally, mechanical loading may influence the orientation of TCR variable and constant domains, interactions within membrane-proximal regions, positioning of transmembrane helices, and the state of associated lipids. However, the precise molecular sequence that couples mechanical force on TCR-pMHC to ITAM exposure has not yet been fully established. Current structural data instead suggest that mechanical loading, ligand-dependent allostery, and the lipid environment are interconnected rather than mutually exclusive mechanisms of TCR regulation.

Taken together, current evidence argues against viewing the TCR as a static receptor switch. A more appropriate model is that of a dynamic structural ensemble in which transition to a signaling-competent state is governed by the combined effects of ligand-induced conformational changes, transmembrane allostery, local lipid remodeling, mechanical forces, and spatial receptor clustering. This has direct implications for TCR-T cell therapy because changes in the transmembrane and membrane-proximal regions of a therapeutic TCR may affect not only receptor expression but also basal activity, antigen sensitivity, and the threshold for signal initiation.

## 4 Mechanisms of signal initiation

Binding of the T-cell receptor to peptide-MHC (pMHC) does not directly generate a second messenger because TCR-CD3 subunits lack intrinsic enzymatic activity. Signal transduction begins with a rapid shift in the balance between membrane-associated tyrosine kinases and phosphatases in the immediate vicinity of the engaged TCR. The key components of this proximal signaling module are the Src-family kinase Lck, CD3 ITAMs, the tyrosine kinase ZAP-70, and several tyrosine phosphatases, most notably CD45 and SHP-1. Their interplay determines not only whether a signal is generated, but also its threshold, amplitude, duration, and the ability of the T cell to discriminate between agonist and non-agonist pMHC ligands (29–32).

### 4.1 Lck as the initiator of TCR-CD3 phosphorylation

Lck (lymphocyte-specific protein tyrosine kinase) occupies a central position in TCR signal initiation by converting extracellular pMHC recognition into an intracellular phosphorylation signal. Lck is a Src-family tyrosine kinase associated with the inner leaflet of the plasma membrane and with the cytoplasmic regions of the CD4 and CD8 coreceptors. Consequently, formation of a TCR-pMHC-CD4/CD8 complex can concentrate Lck in close proximity to the CD3 cytoplasmic domains (29, 30).

The functional state of Lck has traditionally been linked to phosphorylation of two key tyrosine residues. Phosphorylation of Tyr394 (Y394) in the activation loop of the catalytic domain stabilizes an active kinase conformation. In contrast, phosphorylation of the C-terminal Tyr505 (Y505), mediated primarily by Csk, promotes intramolecular binding of pY505 to the Lck SH2 domain and formation of a more closed, catalytically constrained conformation. Dephosphorylation of Y505 by CD45 facilitates transition of Lck to an open signaling-competent state, after which trans-autophosphorylation of Y394 increases catalytic activity (32, 33).

However, the simple model in which inactive Lck becomes activated only after TCR engagement is now considered insufficient. Resting naive T cells already contain a substantial pool of catalytically active Lck; in one seminal study, the active form accounted for up to 40% of total kinase. A fraction of Lck was phosphorylated simultaneously at Y394 and Y505. TCR stimulation did not markedly increase the overall amount of catalytically active Lck, whereas the pre-existing size of this pool determined the efficiency of CD3ζ phosphorylation (34). These findings support a model in which TCR triggering reflects spatial access of pre-existing active Lck to its substrates rather than obligatory *de novo* activation of the kinase.

At the same time, Lck conformation and phosphorylation remain interdependent parameters. Experimental studies indicate that conformational opening and Y394 phosphorylation jointly regulate the ability of Lck to initiate TCR signaling (35). Thus, the functional Lck pool is better described as an ensemble of states with different combinations of regulatory tyrosine phosphorylation, conformational accessibility, and spatial localization rather than as two discrete “active” and “inactive” forms.

### 4.2 Spatial segregation of kinases and phosphatases during signal initiation

One mechanism that explains the extremely rapid transition from pMHC binding to TCR phosphorylation is the kinetic-segregation model. According to this model, formation of the short intermembrane TCR-pMHC complex creates a close-contact zone between the T cell and the antigen-presenting cell. The large extracellular domain of CD45 is sterically incompatible with this short intermembrane distance and is therefore excluded from the vicinity of the ligated TCR. As a result, the local Lck/CD45 activity ratio shifts sharply toward tyrosine kinase activity, enabling accumulation of phosphorylated ITAMs (36–38).

This spatial segregation occurs at the earliest stages of T-cell interaction with an APC, including contacts formed at microvillar tips. Moreover, CD45 is partially excluded from these regions even before antigen recognition, potentially creating areas on the T-cell surface that are primed for TCR signal initiation (39). Upon antigen contact, TCR-CD45 segregation can develop within seconds.

Kinetic segregation should not, however, be regarded as the sole mechanism of receptor triggering. High-affinity ligands can activate TCRs in experimental systems even when CD45 segregation is substantially reduced (40). Spatial exclusion of phosphatases likely acts together with TCR conformational changes, mechanical forces, local receptor clustering, and recruitment of coreceptor-associated Lck.

### 4.3 ITAM phosphorylation

The first direct biochemical targets of Lck after formation of a signaling-competent receptor complex are the immunoreceptor tyrosine-based activation motifs (ITAMs) within the CD3 cytoplasmic domains. An ITAM has the consensus sequence YxxL/I-(x)6–8-YxxL/I. Phosphorylation of both tyrosine residues within a single ITAM creates a high-affinity binding site for the two SH2 domains of ZAP-70 (29, 41).

Lck phosphorylates individual tyrosines within CD3ζ at different rates, so pITAM formation is not fully synchronous. Analysis of individual CD3ζ sites revealed sequential differences in phosphorylation kinetics (42). Thus, individual ITAMs are not functionally equivalent signaling modules: the extent and pattern of their phosphorylation may encode information about the strength and duration of TCR-ligand interactions.

Partial ITAM phosphorylation has different functional consequences from dual phosphorylation. Although some Src-family kinases can interact with monophosphorylated motifs, stable high-affinity recruitment of ZAP-70 predominantly requires phosphorylation of both ITAM tyrosines (43). This creates one of the earliest biochemical checkpoints that prevents a brief stochastic TCR-pMHC encounter from being converted into a full signaling response.

Sequential ITAM phosphorylation can therefore be viewed as an element of kinetic proofreading. If a TCR-pMHC complex dissociates too rapidly, phosphatases can reverse intermediate phosphorylation steps before the state required for stable ZAP-70 binding is reached. Longer engagement by an agonist pMHC increases the probability that successive steps will be completed and a signaling-competent complex will form (30, 31).

The presence of ten ITAMs is a characteristic feature of the TCR-CD3 complex, but their multiplicity should not be viewed solely as a mechanism of linear signal amplification. Experimental evidence indicates that the number of available functional ITAMs can influence the efficiency with which ligand engagement is converted into productive intracellular signaling, the fraction of responding cells, and the synchrony of proximal signal initiation. Thus, in addition to the extent and kinetics of phosphorylation of individual ITAMs, their functional multiplicity provides an additional layer of quantitative TCR signal tuning (44).

### 4.4 Recruitment and activation of ZAP-70

The next obligatory step in canonical TCR signaling is recruitment of the tyrosine kinase ZAP-70 (ζ-chain-associated protein kinase of 70 kDa). In resting cells, ZAP-70 is predominantly cytoplasmic and adopts an autoinhibited conformation. Its N-terminal region contains two tandem SH2 domains that selectively bind doubly phosphorylated ITAMs.

Binding of the tandem SH2 domains of ZAP-70 to a doubly phosphorylated ITAM recruits the kinase to the cytoplasmic region of TCR-CD3 and partially relieves its autoinhibited state. However, pITAM binding alone only modestly increases ZAP-70 catalytic activity. Full activation requires a subsequent phosphorylation step, mediated primarily by Lck (45, 46).

Tyrosine residues Y315 and Y319 in the linker between the C-terminal SH2 domain and the catalytic kinase domain (SH2-kinase linker, interdomain B), together with Y493 in the activation loop of the kinase domain, play key roles in regulating ZAP-70 activity. Phosphorylation of Y319 helps stabilize the active conformation of ZAP-70 and creates a binding site for the SH2 domain of Lck and other signaling components (47). Phosphorylation of Y493 directly increases ZAP-70 catalytic activity and is critical for antigen-receptor signaling (48). Structural studies indicate that pITAM binding and subsequent Lck-mediated phosphorylation act sequentially: the former disrupts part of the autoinhibitory interactions, whereas phosphorylation of regulatory tyrosines produces robust kinase activation.

Thus, the initial stages of TCR signal transduction proceed through a defined sequence of events. Recognition of pMHC by the TCR allows Lck to phosphorylate ITAM motifs in the CD3 subunits, thereby creating binding sites for ZAP-70. Once recruited to the receptor complex, ZAP-70 is phosphorylated by Lck and becomes catalytically active. Active ZAP-70 then phosphorylates LAT and SLP-76, promoting assembly of the LAT-SLP-76 signalosome (30, 45). These events are summarized in **Figure 1**.

**Figure 1:**
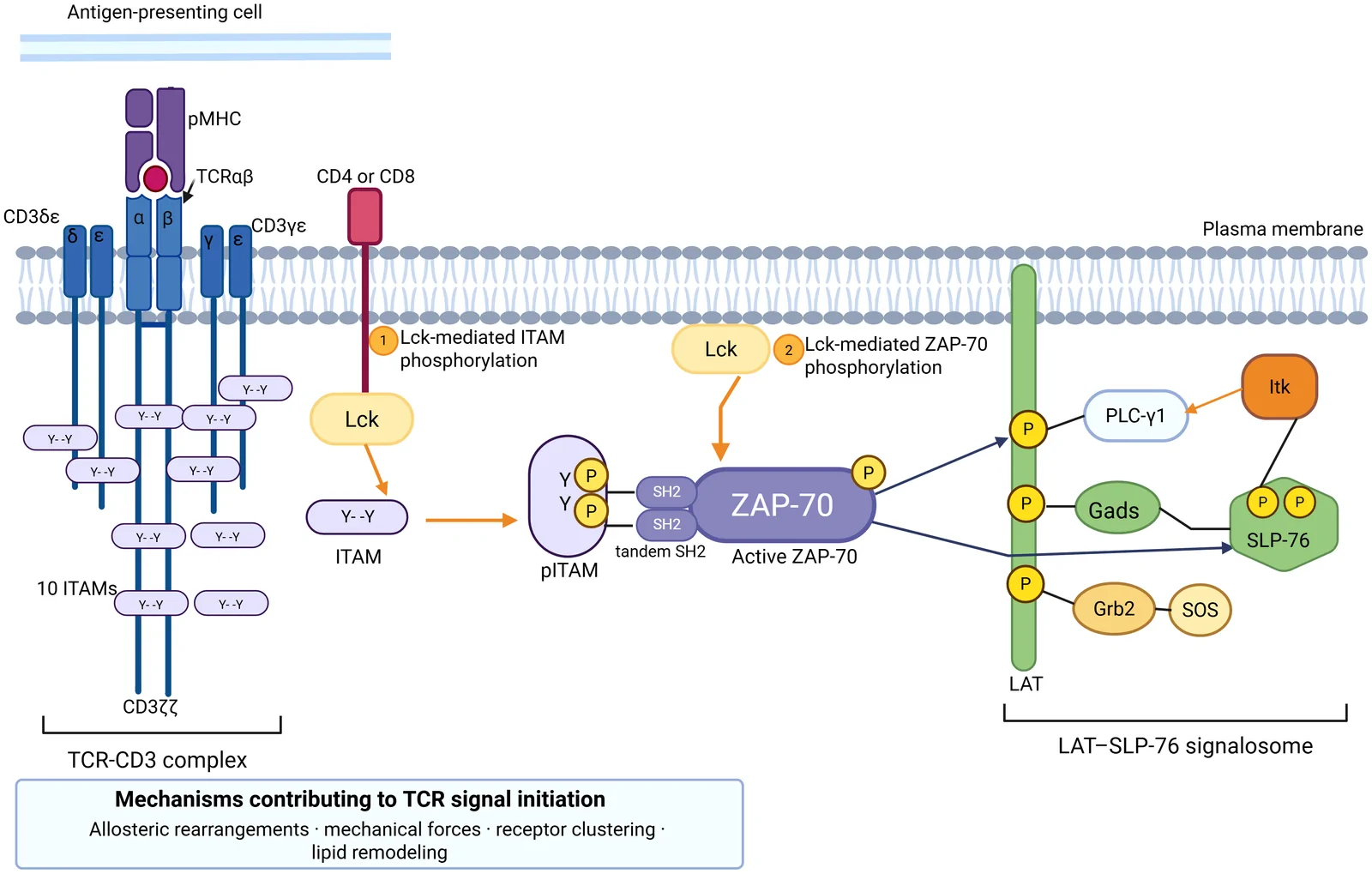
Structural organization of the TCR-CD3 complex and initiation of the proximal TCR signaling cascade. Recognition of pMHC by TCRαβ brings Lck, associated with the CD4 or CD8 coreceptor, into proximity with the signaling chains of the CD3 complex. TCR-CD3 contains ten ITAM motifs: one in CD3γ, one in CD3δ, one in each of the two CD3ϵ chains, and three in each CD3ζ chain. Allosteric rearrangements, mechanical forces, receptor clustering, and local remodeling of the lipid environment facilitate TCR signal initiation by regulating ITAM accessibility and phosphorylation. Lck first phosphorylates tyrosine residues within ITAMs, creating pITAM docking sites for the tandem SH2 domains of ZAP-70. Lck then phosphorylates the recruited ZAP-70 molecule, promoting its activation. Active ZAP-70 phosphorylates LAT and SLP-76, enabling assembly of the LAT-SLP-76 signalosome, which includes Gads, PLC-γ1, Itk, and the Grb2-SOS complex. Y and P denote tyrosine residues and phosphate groups, respectively.

Once activated, ZAP-70 transmits the signal from TCR-CD3 to more distal components of the cascade by phosphorylating primarily the adaptor proteins LAT and SLP-76. This leads to assembly of the multicomponent LAT-SLP-76 signalosome, which serves as a platform for activation of major downstream TCR pathways, including PLCγ1-Ca^2+^-NFAT, Ras-MAPK-AP-1, and PKCθ-NF-κB. At this stage, the local proximal signal generated by TCR-CD3 is therefore converted into a branched intracellular signaling network that determines subsequent T-cell functional responses (29, 45).

### 4.5 LAT-SLP-76 signalosome and spatial organization of signaling

Phosphorylated LAT functions not as a single linear adaptor but as a multivalent membrane platform. Phosphotyrosine sites in LAT recruit Grb2/SOS1 and Gads/SLP-76, while SLP-76 in turn organizes interactions with PLCγ1, Itk, Vav1, Nck, and actin-cytoskeletal proteins. Through multiple low- and moderate-affinity interactions, these molecules assemble into dynamically organized micro- and nanoclusters that spatially couple the proximal TCR signal to Ras-MAPK activation, Ca^2+^-NFAT signaling, and actin remodeling (49, 50).

In a reconstituted membrane system, Su et al. showed that components of the LAT signalosome can form liquid-like condensates through phase separation. These condensates were enriched in kinases and adaptor proteins while restricting access of several phosphatases, thereby creating a local biochemical environment favorable for signal amplification and actin assembly (49). This physical organization complements the TCR-microcluster model: signaling is determined not only by the number of phosphorylated molecules but also by the efficiency of their spatial concentration within signaling domains. Thus, the LAT-SLP-76 signalosome is a dynamically organized molecular platform that can, under appropriate conditions, form condensate-like structures; its properties depend on interaction valency, the degree of phosphorylation, and linkage to the cytoskeleton. Current concepts of TCR-microcluster structure, assembly kinetics, and regulation, including the spatial organization of signaling components and their dynamic exchange, have been synthesized by Balagopalan et al. (51).

CD28 co-stimulation can influence not only later PI3K-Akt-mTOR-dependent processes but also the earliest stages of TCR signaling. Recent data show that CD28 enhances recruitment of Lck to TCR microclusters, accelerates recruitment of ZAP-70 to TCRζ, and increases ZAP-70 activation, thereby altering the kinetics of the proximal signal (52). These findings extend the classical view of CD28 as a predominantly distal co-stimulatory receptor and demonstrate that TCR and CD28 signals are integrated already at the level of the early signaling complex.

These observations also have translational implications because they indicate that altering co-stimulatory modules in engineered T-cell receptor systems may affect not only later survival and metabolic programs but also the kinetics and threshold of the proximal signal.

### 4.6 CD45 as a positive and negative regulator of early TCR signaling

CD45 (PTPRC) is one of the most highly expressed transmembrane tyrosine phosphatases in T cells and provides a particularly clear example of why phosphatases cannot be viewed solely as signal “off switches”.

On the one hand, CD45 has a positive role in preparing T cells for activation. By dephosphorylating the inhibitory Y505 residue of Lck, it prevents accumulation of the closed autoinhibited kinase and maintains a pool of Lck that can rapidly participate in TCR signaling (32, 33). Loss or very low activity of CD45 leads to accumulation of pY505-Lck and profound defects in TCR signaling.

On the other hand, CD45 can dephosphorylate the activating Y394 residue of Lck as well as phosphorylated TCR components. Excessive CD45 in the immediate vicinity of an engaged receptor therefore prevents pITAM accumulation. Experimental manipulation of CD45 expression revealed a nonlinear effect: very low phosphatase activity suppressed TCR signaling, intermediate activity could produce hyperresponsiveness, whereas high physiological expression constrained Lck activity. These observations led to the concept of CD45 as a regulatory “rheostat” that sets the TCR activation threshold through differential control of Y505 and Y394 phosphorylation in Lck (33, 53).

### 4.7 SHP-1 and negative feedback

The cytoplasmic tyrosine phosphatase SHP-1 (Src homology region 2 domain-containing phosphatase-1; PTPN6) functions predominantly as a negative regulator of TCR signaling. It contains two SH2 domains through which it can be recruited to phosphorylated signaling complexes.

ZAP-70 is one direct target of SHP-1. Following TCR stimulation, SHP-1 can associate with ZAP-70; SHP-1 activity increases while ZAP-70 catalytic activity decreases. Expression of dominant-negative SHP-1 increases T-cell sensitivity to stimulation, supporting a role for SHP-1 as one determinant of the TCR activation threshold (54).

Regulation of Lck provides another important mechanism. Interaction of the TCR with weak or antagonistic ligands can rapidly engage an SHP-1-dependent negative-feedback loop that reduces Lck activity and desensitizes the receptor complex. In contrast, strong agonists activate ERK-dependent positive feedback; modification of Lck limits effective SHP-1 engagement and sustains signaling (55). Thus, SHP-1 participates not only in terminating an established signal but also directly in ligand discrimination at the earliest stages of TCR activation.

These mechanisms allow weak, short-lived TCR interactions with most self-pMHC complexes to return rapidly to baseline, whereas an agonist signal that crosses a critical threshold can be amplified through positive-feedback mechanisms.

### 4.8 SHP-2: context-dependent regulation of TCR signaling

The role of the related phosphatase SHP-2 (PTPN11) is considerably more complex. It is often grouped with SHP-1 as a negative regulator of TCR signaling, but such a designation is an oversimplification.

Genetic studies have shown that SHP-2 can exert a positive function under some conditions: deletion of PTPN11 in thymocytes reduces pre-TCR and TCR signaling and decreases ERK activation (56). SHP-2 is therefore not a constitutive universal inhibitor of TCR signaling.

The best-established negative function of SHP-2 occurs after its recruitment by inhibitory receptors, particularly PD-1. Following PD-1 engagement by PD-L1, tyrosine motifs in the PD-1 cytoplasmic domain become phosphorylated and recruit SHP-2. In this context, activated SHP-2 locally dephosphorylates components of the activating signaling complex. Biochemical reconstitution further showed that CD28 is a preferred target of PD-1-SHP-2 relative to TCR-CD3 itself, substantially revising the original model in which PD-1 was thought to suppress the TCR directly (57).

### 4.9 Dynamic kinase-phosphatase balance sets the T-cell activation threshold

Taken together, early TCR signaling is better viewed not as a linear series of irreversible reactions but as a dynamic balance between kinase- and phosphatase-mediated processes. Lck promotes phosphorylation of CD3 ITAMs and subsequent recruitment and activation of ZAP-70, whereas CD45, SHP-1, and other phosphatases continuously oppose these phosphorylation events and thereby regulate signal amplitude, duration, and threshold.

Even resting T cells display a degree of basal Lck activity and low-level phosphorylation of TCR components. Phosphatase activity, however, prevents signal accumulation above the functional threshold. Agonist pMHC binding changes the spatial organization of this equilibrium: the TCR is retained within the contact zone, local CD45 phosphatase activity decreases, encounters with active Lck become more likely, doubly phosphorylated ITAMs accumulate, and ZAP-70 is recruited. Once the threshold is crossed, positive-feedback mechanisms help stabilize the signal, whereas SHP-1 and other phosphatases constrain its amplitude and duration.

This regulatory system is particularly important for TCR-T cell therapy. The functional activity of an introduced therapeutic TCR is determined not only by its affinity for tumor pMHC and its surface expression level. The effective response threshold is also shaped by the abundance and spatial organization of Lck, CD45, and SHP phosphatases, the availability of CD4/CD8 coreceptors, and the strength of inhibitory signaling. Thus, two TCRs with similar biochemical affinity may generate different cellular responses because of differences in the efficiency of proximal signal propagation. Conversely, excessive reduction of phosphatase control can impair discrimination of weak ligands and enhance tonic signaling. Optimization of therapeutic TCRs should therefore consider not only TCR-pMHC binding parameters but also the kinase-phosphatase balance that determines functional avidity and the activation threshold of TCR-T cells.

Recent studies, including work from our group, indicate that computationally predicted TCR affinity should not be used as an independent criterion for selecting a therapeutic clonotype. During selection of a MAGE-A3-specific TCR, the dominant clonotype had an intermediate predicted ERGO-II binding score, yet transfer of this receptor into T cells produced marked cytotoxicity against MAGE-A3-positive tumor cells (58). In a subsequent comparative study of three HER2/neu-specific TCRs recognizing the same KIFGSLAFL epitope in HLA-A*02, pronounced clonotype-dependent functional heterogeneity was again observed and could not be reduced to simple ranking by predicted affinity (59). Thus, in silico binding estimates may be useful for preliminary candidate screening but cannot replace functional validation and are not equivalent to an experimentally determined dissociation constant (K_D_).

### 4.10 Establishment of TCR signaling thresholds during thymopoiesis

The signaling competence of a mature T cell is established in part during thymic selection. In CD4+CD8+ thymocytes, the strength and duration of TCR interactions with self-pMHC determine alternative selection outcomes: an insufficient signal fails to support survival, signals within an intermediate range support positive selection, whereas stronger recognition of self ligands promotes negative selection. Not only amplitude but also the temporal and spatial organization of signaling is important, including differences in Ras-MAPK/ERK dynamics during positive versus negative selection (60, 61). Thus, already in the thymus, the TCR functions as a quantitative sensor of the strength and duration of pMHC engagement.

These selection thresholds are influenced by the state of the thymocyte and by the thymic microenvironment. Metabolic programs differ across developmental stages and can alter the capacity of a cell to sustain proliferation, survival, and TCR-dependent signaling responses (62). In addition, pattern-recognition receptor signals in cells of the thymic microenvironment, including responses to PAMPs/MAMPs and DAMPs, can alter the cytokine milieu, the state of antigen-presenting cells, and the conditions under which self antigens are presented (63). Thus, formation of the peripheral TCR repertoire is determined not only by TCR structure and affinity for pMHC but also by the metabolic and inflammatory context in which positive and negative selection occur.

## 5 Metabolic constraints as modifiers of the TCR signal

Following TCR and CD28 activation, T cells increase glucose and amino-acid transport and activate MYC-, PI3K-Akt-mTOR-, and HIF-1α-dependent programs (64–69). This does not represent a complete switch from oxidative phosphorylation to glycolysis: mitochondrial metabolism is retained and simultaneously supports biosynthesis, redox homeostasis, and early signaling events (64, 70–75). Amino-acid availability provides an additional layer of metabolic control, as glutamine uptake and serine availability contribute to anabolic growth and T-cell proliferation (68, 69, 76). Accordingly, in the context of this review, metabolism is considered primarily as a modifier of receptor signaling rather than as an independent parallel program.

The link to TCR signaling is particularly evident for glucose metabolism. Glucose availability supports IFN-γ translation, whereas phosphoenolpyruvate (PEP) limits SERCA activity and thereby sustains Ca^2+^-NFAT signaling; when glucose is limited, this component of the TCR-dependent response is attenuated (77, 78). Mitochondrial reactive oxygen species generated by the respiratory chain are also required for efficient early activation following TCR engagement (75). Thus, metabolic substrates influence not only the energetic support of effector function but also the efficiency of receptor-signal propagation itself.

Within tumors, T cells compete with transformed cells for glucose and amino acids while simultaneously experiencing hypoxia and exposure to immunosuppressive metabolites. Metabolic competition can reduce IFN-γ production and weaken the TCR-dependent effector program even when antigen recognition is preserved, as illustrated in **Figure 2** (79). This factor is particularly important for TCR-T cells during repeated stimulation: even a functionally potent receptor does not guarantee durable activity if the cell cannot maintain mitochondrial plasticity and biosynthetic capacity under nutrient-limited conditions. Consistent with this concept, antigen recognition by NY-ESO-1-specific TCR-T cells in a 3D tumor model was accompanied by increased oxygen consumption and enhanced mitochondrial ATP production, providing direct evidence that target recognition is coupled to metabolic adaptation in engineered T cells (80).

**Figure 2:**
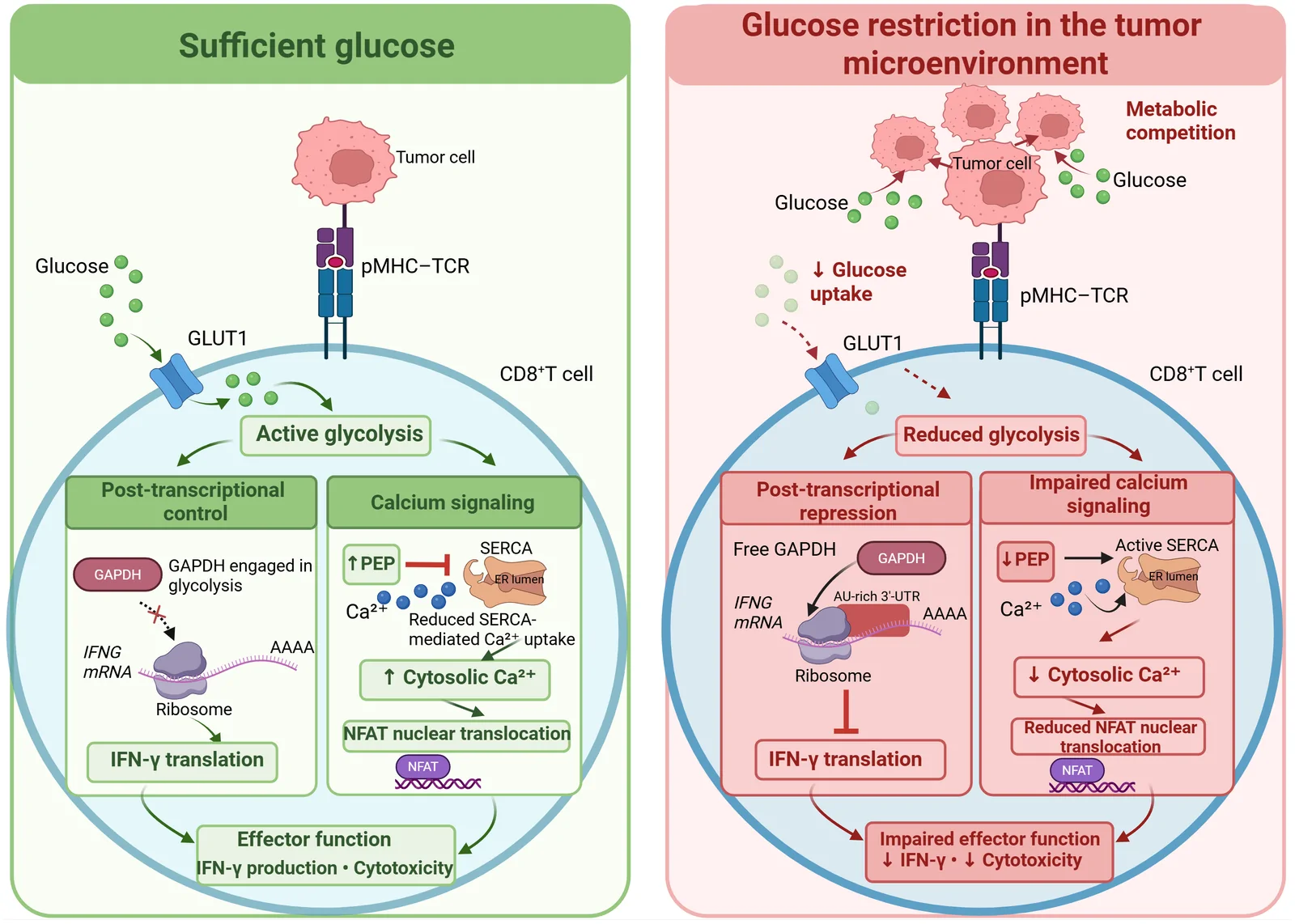
Glucose availability controls TCR-dependent effector function in the tumor microenvironment. Under glucose-replete conditions, glucose uptake through GLUT1 maintains active glycolysis in antigen-stimulated CD8+ T cells. When glycolytic flux is high, GAPDH is engaged in glycolytic reactions and is therefore less available to bind IFNG mRNA, permitting efficient IFN-γ translation. Glycolysis also generates PEP, which limits SERCA activity and Ca^2+^ uptake into the ER, thereby sustaining cytosolic Ca^2+^ levels and NFAT activation. In the tumor microenvironment, increased glucose consumption by malignant cells can restrict glucose availability to tumor-reactive T cells and reduce their glycolytic activity. Under these conditions, GAPDH becomes available to bind the AU-rich region within the 3′-UTR of IFNG mRNA and suppress its translation. Simultaneously, reduced PEP levels permit greater SERCA activity, promoting Ca^2+^ sequestration in the ER, decreasing cytosolic Ca^2+^ availability, and attenuating NFAT nuclear translocation. Together, these mechanisms reduce IFN-γ production and cytotoxic activity despite antigen recognition through pMHC–TCR and may limit the functional activity of tumor-reactive T cells, including TCR-T cells.

In practical terms, metabolic fitness should be assessed together with TCR-pMHC interaction parameters and repeated-stimulation responses when therapeutic T cells are characterized. However, the detailed biochemistry of immunometabolism lies beyond the scope of this review and is considered here only insofar as it modifies the threshold, duration, and functional outcome of TCR signaling (70, 72, 81, 82).

## 6 Functional outcomes of TCR signaling: effector responses, AICD, and exhaustion

Recognition of pMHC by the TCR initiates transcriptional, epigenetic, and metabolic changes that depend on signal strength and duration, antigen density, coreceptors, co-stimulation, cytokines, and metabolic state. During chronic antigen exposure, a fraction of cells enters a state of T-cell exhaustion (TEX), characterized by altered effector functions, sustained co-expression of inhibitory receptors, and a distinct transcriptional and epigenetic program (83–85).

This process is particularly important in TCR-T cell therapy. An introduced tumor-specific TCR may confer high sensitivity to the target pMHC, but persistent tumor antigen generates chronic TCR signaling that progressively changes the functional state of the engineered cell. TCR-T efficacy therefore depends not only on the ability to recognize the tumor and execute an initial cytotoxic response, but also on the capacity to preserve function and proliferative reserve during repeated and prolonged antigenic stimulation (86–88). The major determinants of T-cell fate as a function of TCR signal strength, duration, co-stimulation, and chronic antigen exposure are summarized in **Figure 3**.

**Figure 3:**
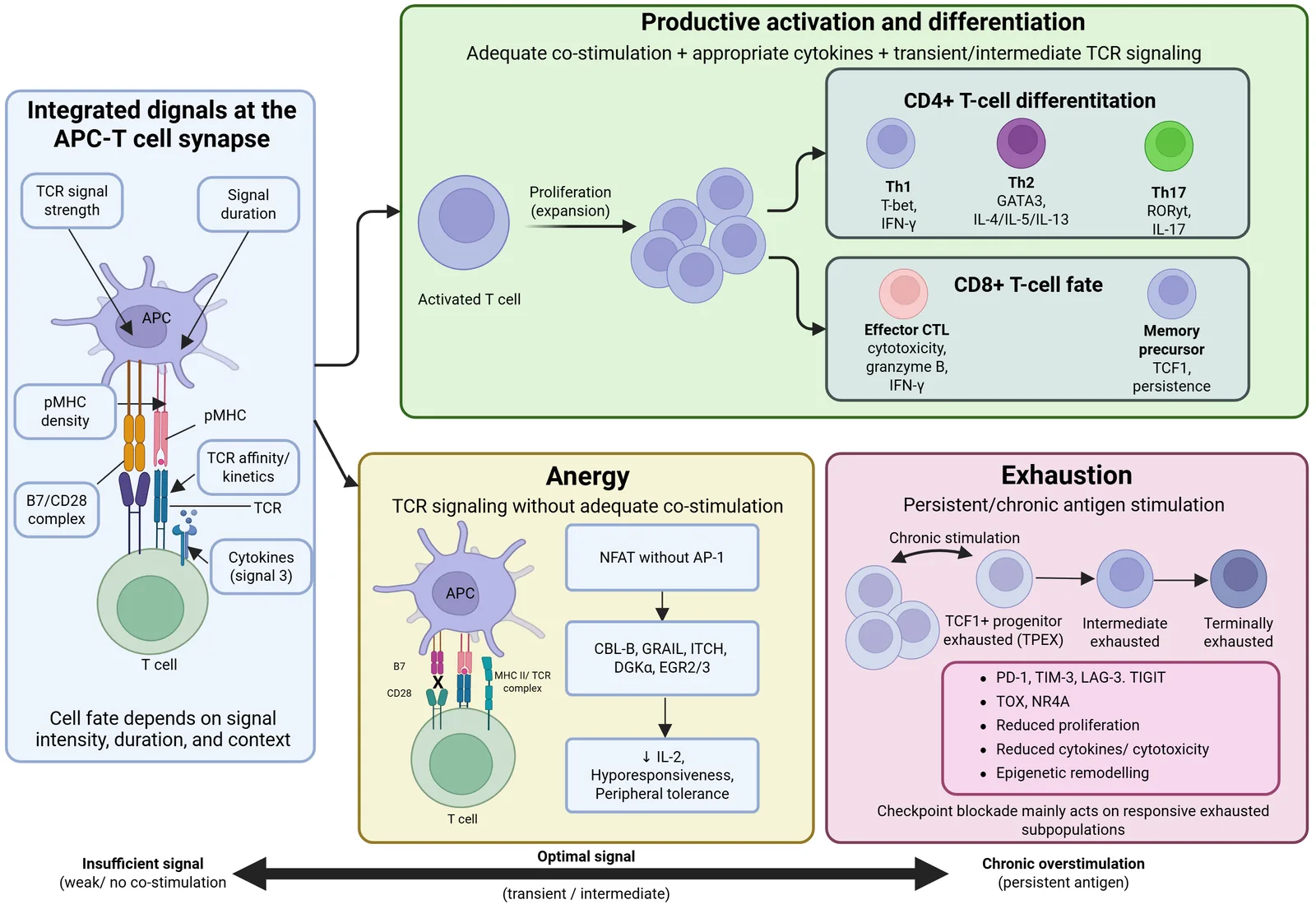
Integrated TCR signaling determines T-cell functional fate. The combined effects of TCR-pMHC affinity and kinetics, pMHC density, signal strength and duration, co-stimulation, and cytokine input shape T-cell responses. Transient or intermediate signaling in the presence of adequate co-stimulation promotes productive activation, clonal expansion, effector differentiation, and memory formation. TCR signaling in the absence of sufficient co-stimulation can favor anergy, whereas persistent antigenic stimulation drives progressive T-cell exhaustion, including transition from TCF1+ progenitor exhausted (TPEX) cells through intermediate states to terminally exhausted cells. Thus, T-cell fate is determined by the integrated magnitude and duration of TCR signaling rather than by receptor affinity alone. A separate outcome of excessively strong or repeated stimulation is AICD, which is mechanistically distinct from exhaustion and involves Fas/FasL-FADD-caspase-8- and BIM-dependent cell-death pathways (89, 90).

### 6.1 Establishment of the effector program after TCR activation

During full activation, the proximal TCR-Lck-ZAP-70 cascade drives formation of the LAT-SLP-76 signalosome and parallel activation of three major transcriptional axes: Ca^2+^-calcineurin-NFAT, Ras-MAPK-AP-1, and PKCθ-NF-κB. Their coordinated activity converts the initial receptor signal into a gene-expression program required for cell growth, proliferation, and acquisition of effector properties (29, 91).

Cooperative action of NFAT and AP-1 is particularly important. NFAT is activated by increased intracellular Ca^2+^ and calcineurin-dependent dephosphorylation, whereas AP-1, composed mainly of FOS- and JUN-family proteins, is induced downstream of Ras-MAPK signaling. NFAT-AP-1 complexes bind regulatory regions of numerous activation genes, including IL-2 and other components of the effector program. Full TCR signaling therefore depends not simply on activation of individual transcription factors but on their coordinated integration (91).

In CD8+ T cells, acquisition of an effector phenotype is accompanied by expression of the cytotoxic molecules perforin and granzymes, increased production of IFN-γ and TNF, changes in chemokine receptors and adhesion molecules, and enhanced migration to inflamed tissues. When a TCR encounters a sufficient number of cognate pMHC complexes, an immunological synapse forms, the microtubule-organizing center and secretory lysosomes polarize toward the target cell, and cytotoxic granules undergo directed exocytosis.

### 6.2 TCR affinity, antigen density, and integrated receptor signal strength

The functional response of a T cell is determined not by any single parameter of its interaction with a target cell but by the integrated strength of TCR signaling. Major determinants include the affinity and kinetics of TCR binding to peptide-MHC (pMHC), the density of cognate pMHC on the target-cell surface, TCR expression on the T cell, participation of CD4/CD8 coreceptors, and duration of cell-cell contact. The same TCR can therefore induce markedly different functional responses depending on the amount of cognate pMHC presented by the target cell (88, 92).

Comparison of three HER2/neu-specific TCRs recognizing KIFGSLAFL/HLA-A*02 illustrates why function cannot be reduced to a single binding parameter. ERGO-II affinity scores were 0.72, 0.83, and 0.55 for constructs 1–3, respectively, yet construct 3, which had the lowest predicted score, mediated the strongest cytotoxicity *in vitro* and the most pronounced tumor regression *in vivo* (59). Multimodal profiling confirmed an enhanced cytolytic and cytokine program in this construct.

For TCR-T cells, expression of the source tumor protein must be distinguished from the density of a specific pMHC complex. The latter depends on antigen processing, peptide transport and loading, peptide-HLA stability, and HLA expression; it is the number of accessible cognate pMHC complexes that directly determines the number of potential productive TCR interactions (88).

### 6.3 TCR affinity and sensitivity to low pMHC density

Within the physiological range, increasing TCR affinity for pMHC generally increases antigen sensitivity. A higher-affinity receptor can support productive interactions at lower pMHC abundance and initiate early signaling more readily. Increasing TCR affinity has therefore historically been viewed as a strategy to improve recognition of tumor cells, on which tumor-specific epitopes are often displayed at low density (93–95).

However, the relationship between affinity and function is nonlinear. Studies of tumor-antigen-specific TCRs have shown that increasing affinity within the physiological range improves antigen sensitivity and polyfunctionality, whereas further increases into a supraphysiological range cease to provide functional benefit and may even reduce activity (94–96).

For example, studies of a panel of HLA-A2/NY-ESO-1-specific TCRs identified a functional affinity window: T cells expressing receptors of intermediate-to-high affinity showed optimal function, whereas further affinity increases were associated with signaling defects, increased SHP-1 activity, and, for the highest-affinity receptors, marked PD-1 expression (94). Thus, affinity above a certain threshold may constrain rather than enhance functional responses.

Excessive artificial affinity enhancement may also alter antigen-density requirements and recognition specificity. For some affinity-matured TCRs, supraphysiological affinity unexpectedly failed to improve recognition of cells displaying very low densities of the original pMHC (97). Biophysical TCR affinity therefore cannot be used as the sole indicator of therapeutic efficacy.

### 6.4 pMHC density as an independent regulator of T-cell responses

The number of cognate pMHC complexes on a target cell is an independent variable that determines the probability of repeated TCR engagement, the number of receptors engaged simultaneously, and the integrated duration of TCR signaling. CD8+ T cells can be extraordinarily sensitive: in some experimental systems, productive responses can be induced by tens or even single cognate pMHC molecules. However, the minimum effective density depends strongly on the particular TCR, binding kinetics, CD8 involvement, the effector readout, and activation conditions (92).

At low pMHC density, the lifetime of the TCR-pMHC complex becomes especially important. In a classic study, González et al. showed that the binding kinetics required for productive T-cell activation depend directly on ligand density on the antigen-presenting cell: when pMHC is scarce, the optimal window of interaction duration is much narrower, whereas increasing ligand density can partially compensate for otherwise unfavorable binding parameters (92).

### 6.5 Cumulative TCR signal strength

Functionally, TCR affinity and pMHC density can be regarded as two components of a common system that determines the cumulative strength of TCR signaling. This integrated signal reflects the combined effects of TCR-pMHC affinity and binding kinetics, the density of cognate pMHC complexes, TCR abundance on the T-cell surface, and the duration of cell-cell contact.

If the integrated signal is too weak, the T cell does not reach the threshold required for effective tumor-cell lysis. At an intermediate signal strength, cytotoxicity, cytokine production, and proliferation are optimally balanced. Excessively strong and especially chronic stimulation increases the probability of entering programs of dysfunction and exhaustion.

This nonlinear relationship was convincingly demonstrated by Shakiba et al. in a tumor model. Strong TCR signaling induced inhibitory receptor expression, loss of effector function, and a dysfunctional program. In contrast, when signaling was too weak, cells retained intrinsic effector potential but remained functionally inert within the tumor and failed to control tumor growth. Reducing excessive TCR signaling to an intermediate level enhanced antitumor activity (98). These data support the existence of an optimal range of TCR signal strength that balances insufficient activation against chronic hyperstimulation.

### 6.6 Effect of affinity on the kinetics of exhaustion

Receptor affinity influences not only initial T-cell sensitivity but also the rate of subsequent differentiation during persistent antigen exposure. In the tumor microenvironment, T cells with stronger TCR-pMHC binding tend to enter the cell cycle more rapidly and progress more quickly toward terminally exhausted states.

Hay et al. showed that, within a polyclonal response to a single tumor-associated antigen, stronger TCR-pMHC binding was associated with enrichment of proliferative cell states and more rapid progression toward terminal exhaustion. In contrast, T cells with weaker binding were more likely to retain a progenitor-like phenotype. At early stages, however, higher TCR affinity was associated with more effective tumor control (99).

### 6.7 High antigen burden as a driver of exhaustion

T-cell functional state is determined not only by the properties of an individual TCR-pMHC interaction but also by the overall magnitude and duration of antigenic stimulation. Sustained exposure to a high antigen burden can promote an exhausted phenotype even in functionally active T cells with high-affinity TCRs.

This was particularly clearly demonstrated by Tay et al. using a model of controlled antigen expression *in vivo*. At relatively low antigen burden, high-affinity CD8+ T cells retained effector function. High expression of the same antigen, however, drove the cells into functional exhaustion. Antigen expression level was a stronger determinant of cell fate than TCR affinity (100).

### 6.8 Impact of heterogeneous antigen expression within tumors

Spatial and clonal tumor heterogeneity represents an additional challenge for TCR-T therapy. Even when a target antigen is present, its expression and the abundance of the corresponding pMHC can vary substantially among individual tumor cells.

This creates opposing selective pressures. Cells with high target-pMHC density are efficiently recognized by TCR-T cells but can also induce the strongest chronic stimulation. Cells with low pMHC density pose the opposite problem: they may generate a signal below the threshold required for effective killing and thereby provide a reservoir for immune escape.

### 6.9 Repeated TCR stimulation and activation-induced cell death

Repeated or excessively strong TCR stimulation can lead not only to functional dysfunction but also to activation-induced cell death (AICD). In previously activated T cells, a classical mechanism of AICD is induction of Fas (CD95)/FasL followed by signaling through FADD and caspase-8. In addition, repeated activation can reduce cell survival through the intrinsic mitochondrial apoptotic pathway, in which BIM is involved (89, 90).

AICD is fundamentally distinct from exhaustion: in the former, the endpoint is elimination of the cell, whereas exhaustion is a persistent functional and transcriptional-epigenetic state of viable cells during prolonged antigen exposure. This distinction is important for therapeutic T cells because excessive amplification of receptor signaling may reduce long-term efficacy both through loss of cells and through accelerated development of dysfunctional states.

### 6.10 Chronic antigenic stimulation as a major driver of exhaustion

Unlike an acute immune response, in which antigen disappears after elimination of an infection or target cell, tumors can sustain TCR stimulation for weeks, months, or longer. Persistent antigen encounter is one of the major factors driving CD8+ T cells into an exhausted state (83–85).

Exhaustion develops progressively: IL-2 production and proliferative reserve decline first, followed by potential reductions in TNF, IFN-γ, and cytotoxicity, while expression of PD-1^high^, TIM-3, LAG-3, TIGIT, and CD244 becomes sustained. No single marker is specific by itself; exhaustion is defined by the combination of functional, transcriptional, and epigenetic features (83, 84).

Accordingly, the term “exhaustion” should be applied to TCR-T cells only when a constellation of features is present, rather than on the basis of PD-1 expression alone. Increased PD-1 within hours of tumor-cell contact primarily reflects activation, whereas a sustained PD-1^high^ TIM-3+ LAG-3+ phenotype after repeated stimulation, together with reduced cytokine responses and proliferation, is far more indicative of progressive dysfunction.

### 6.11 PD-1 and negative regulation of TCR signaling

PD-1 is the best-characterized receptor limiting the functional activity of chronically stimulated T cells. Following engagement by PD-L1 or PD-L2, tyrosine motifs in its cytoplasmic domain become phosphorylated, enabling recruitment of SHP phosphatases and suppression of activating signaling complexes.

As noted above, PD-1 is not a simple TCR off switch: during co-stimulation, CD28 is an important target of the PD-1-SHP-2 complex. PD-1-dependent coinhibition therefore constrains co-stimulation, metabolic activity, cytokine production, and proliferation (57). This mechanism is particularly relevant to TCR-T cells because the natural TCR, unlike most CAR constructs, does not contain an integrated co-stimulatory domain. Persistent TCR signaling combined with insufficient physiological co-stimulation in the tumor microenvironment may therefore contribute to chronic dysfunction.

### 6.12 NFAT-AP-1 imbalance as a molecular transition from activation to exhaustion

One of the key conceptual mechanisms linking chronic TCR signaling to the exhaustion program is a change in the balance between NFAT and AP-1 activity.

During acute activation, NFAT cooperates with AP-1 to support effector genes. During prolonged stimulation, NFAT activity can persist despite insufficient AP-1, thereby driving an alternative transcriptional profile; an NFAT1 variant unable to interact with AP-1 induced a program resembling CD8+ T-cell exhaustion (91).

### 6.13 TOX as a central regulator of the exhaustion program

A major transcriptional consequence of chronic NFAT signaling is increased expression of TOX (thymocyte selection-associated high mobility group box). Several independent studies published in 2019 established TOX as a central regulator of the transcriptional and epigenetic program of exhausted CD8+ T cells (101–104).

Khan et al. showed that TOX is induced through a calcineurin-NFAT2-dependent mechanism during prolonged TCR stimulation. Once induced, TOX promotes the characteristic chromatin-accessibility landscape of TEX cells and supports expression of exhaustion-associated genes. Loss of TOX prevents normal establishment of this transcriptional and epigenetic state (102).

TOX has a dual function: reducing its activity may transiently enhance effector responses, but cells unable to establish a normal TOX-dependent adaptive program show poorer persistence under chronic stimulation (103). The biological role of the TOX program therefore extends beyond suppression of antitumor responses. It represents an adaptation to chronic TCR signaling that reduces the risk of excessive activation and cell death at the cost of diminished effector function. This is highly relevant to TCR-T cell engineering: complete TOX blockade could increase short-term activity while simultaneously reducing long-term persistence.

### 6.14 NR4A factors as an additional component of TCR-induced dysfunction

Another group of transcription factors closely linked to chronic TCR signaling comprises the nuclear receptors NR4A1, NR4A2, and NR4A3. Their expression is also induced by NFAT-dependent signaling.

In an antitumor CAR-T cell model, Chen et al. showed that cells within tumors acquire an NR4A-dependent transcriptional program resembling that of endogenous exhausted TILs. Simultaneous deletion of Nr4a1, Nr4a2, and Nr4a3 markedly improved the antitumor activity of engineered T cells, increased effector-molecule production, and promoted tumor regression (105).

TOX/TOX2 and NR4A form partially overlapping regulatory networks that are activated in response to chronic TCR stimulation. Collectively, available data indicate that persistent TCR signaling sustains Ca^2+^/calcineurin-dependent NFAT activity, which in turn promotes induction of TOX-family and NR4A transcription factors. These regulators contribute to the epigenetic and transcriptional stabilization of the exhausted T-cell (TEX) program (105, 106). During acute activation, NFAT activity is coordinated with AP-1 and other factors that support effector differentiation, whereas during chronic stimulation this balance progressively shifts toward TOX/NR4A-associated transcriptional programs.

### 6.15 Exhaustion as an epigenetically stabilized state

One of the most important advances in recent years has been recognition that exhausted T cells differ from effector and memory cells not only in expression of individual genes but also in chromatin architecture.

ATAC-seq analysis showed that TEX cells acquire a characteristic set of accessible regulatory regions that differs substantially from those of memory and effector CD8+ T cells. These include specific regulatory elements of inhibitory-receptor genes such as PDCD1 (107).

This helps explain why removal of an inhibitory signal does not necessarily restore a terminally exhausted cell to a fully functional memory state. Pauken et al. showed that PD-1 blockade can transiently increase transcriptional and proliferative activity of TEX cells but does not produce comprehensive reprogramming of the established epigenome. After treatment is withdrawn, a substantial fraction of cells reacquires the characteristic exhausted state (108).

Immune checkpoint blockade is therefore more appropriately viewed not as complete reversion of terminally exhausted cells to a memory state, but as functional enhancement of subsets that retain therapeutic responsiveness. In the context of TCR-T therapy, this supports strategies aimed at preventing stable epigenetic fixation of dysfunction.

### 6.16 Exhausted T cells as a heterogeneous system

The term “exhausted T cell” encompasses several functionally and biologically distinct states. Particularly important was the identification of TCF1+ PD-1+ progenitor/stem-like exhausted T cells (TPEX). TPEX cells retain TCF1 expression, self-renewal capacity, and high proliferative potential and serve as an important source of cells that respond to immune checkpoint blockade (109, 110). In tumors, TCF1+PD-1+ CD8+ T cells can self-renew and generate more differentiated progeny; this population accounts for a substantial fraction of the proliferative response to checkpoint blockade (111).

In simplified terms, chronic T-cell differentiation can be viewed as a continuum that begins with TCF1+ progenitor exhausted (TPEX) cells, progresses through intermediate exhausted states, and culminates in TCF1− terminally exhausted (TEX-term) cells. TPEX cells have lower immediate cytotoxicity but a greater capacity to sustain the response, whereas terminally exhausted cells retain some effector function but show reduced self-renewal and weaker therapeutic responsiveness (112).

Importantly, development of a TEX phenotype should not be regarded exclusively as an adverse consequence of chronic TCR stimulation. Under persistent antigen exposure, continuously maximal effector activity would increase the risk of activation-induced cell death and tissue damage. TOX-dependent restriction of the effector program may therefore serve an adaptive function by supporting the long-term maintenance of a fraction of antigen-specific T cells (85, 103).

This consideration is particularly relevant to the design of engineered T-cell therapies. A strategy aimed at completely eliminating exhaustion-associated mechanisms could theoretically enhance the initial effector response while simultaneously increasing the risk of rapid depletion of the cellular pool, including through activation-induced cell death. A more rational goal is therefore to preserve a balance among effector activity, resistance to chronic stimulation, self-renewal capacity, and long-term persistence.

Metabolic constraints within the tumor microenvironment can further shift this balance by limiting the ability of chronically stimulated T cells to sustain their functional state, as discussed above in the context of metabolic modulation of TCR signaling (86, 113, 114).

### 6.17 Implications for therapeutic TCR selection and functional evaluation

For TCR-T therapy, it is essential to evaluate not maximal biochemical receptor affinity per se but the range of conditions under which the receptor provides an effective and durable functional response. Selection of a therapeutic TCR should therefore include parallel characterization of TCR-pMHC affinity and kinetics, functional avidity of TCR-T cells, expression of the transgenic TCR, CD4/CD8 dependence of recognition, the minimum pMHC density required for activation, and the relationship between pMHC density and cytotoxicity or cytokine production (88, 92–100).

For each therapeutic TCR, a curve relating pMHC density to functional response is particularly informative when lysis, IFN-γ/TNF/IL-2 production, and degranulation are assessed in parallel. A single measurement of effector function is insufficient: TCR-T cells should additionally be tested in models of repeated antigenic stimulation, with assessment of preserved proliferation and cytotoxicity, AICD, cytokine production, expression of PD-1, TIM-3, LAG-3, and TIGIT, TOX/NR4A dynamics, the ratio of TCF1+ to TCF1- states, and, where appropriate, metabolic function (85–88, 91–115).

This approach allows determination not only of EC50 or a minimum sensitivity threshold but also of the functional signaling window within which a TCR can recognize cells displaying low pMHC density while preserving viability, proliferative reserve, and resistance to repeated antigenic stimulation. Optimization of TCR-T therapy should therefore seek not to maximize receptor signal strength but to achieve a range that provides the optimal balance among sensitivity, effector activity, and long-term cellular persistence.

## 7 Discussion and therapeutic perspectives on TCR signaling

Current evidence characterizes TCR signaling as a multilevel system in which the outcome is determined by receptor spatial organization, TCR-pMHC affinity and kinetics, pMHC density, kinase and phosphatase activity, co-stimulation, metabolism, and the duration of antigenic stimulation (29–31, 116).

The TCR does not transmit binary information about antigen presence; instead, it generates quantitatively and qualitatively distinct signals that can lead to effector differentiation, memory, anergy, tolerance, or exhaustion (29–31).

These mechanisms have direct translational relevance: in autoimmune disease, therapeutic strategies seek to raise the threshold for pathological T-cell activation and restore tolerance, whereas in oncology they aim to reduce inhibition of tumor-specific cells or confer defined antigen specificity (116).

### 7.1 TCR signaling as a system of dynamic balance

A major conclusion of current research is that no single molecule or reaction uniquely determines the threshold for TCR activation. Rather, this threshold emerges from a dynamic balance among multiple processes.

TCR-pMHC contact increases access to Lck and promotes accumulation of pITAMs, ZAP-70, and the LAT-SLP-76 signalosome, whereas CD45, SHP-1, and SHP-2 constrain signal amplitude and duration. CD28 supports PI3K-Akt-mTOR-dependent co-stimulation, while CTLA-4 and PD-1 act in the opposite direction (29–31, 57).

At later stages, the outcome depends strongly on the duration of receptor stimulation. Time-limited signaling promotes effector-cell and memory formation; TCR signaling in the absence of adequate co-stimulation can induce anergy; and persistent signaling in the continued presence of antigen favors an NFAT-TOX/NR4A-dependent exhaustion program (83, 91, 99, 102, 105, 116).

In simplified terms, the overall functional outcome of TCR signaling is determined by the combined effects of TCR structure, TCR-pMHC affinity and binding kinetics, pMHC density, co-stimulatory and co-inhibitory signals, the kinase–phosphatase balance, cellular metabolism, and the duration of stimulation. Together, these factors shape the integrated strength and temporal profile of TCR signaling, thereby influencing whether T cells undergo activation, differentiation, memory formation, anergy, or exhaustion.

### 7.2 Suppressing pathological T-cell activation in autoimmune disease

In autoimmune disease, the therapeutic objective is not necessarily complete suppression of T-cell function, but rather elevation of the activation threshold of autoreactive cells or restoration of tolerance.

One of the most successful examples of translating fundamental TCR-signaling biology into therapy is blockade of CD28 co-stimulation.

Full activation of a naive T cell requires interaction of CD28 with CD80/CD86 on the antigen-presenting cell. In the absence of this signal, isolated pMHC recognition through the TCR can lead to functional anergy. This principle was exploited in the development of CTLA4-Ig, a soluble form of the extracellular region of CTLA-4 fused to an immunoglobulin Fc fragment.

Abatacept binds CD80/CD86 and prevents CD28-dependent co-stimulation; the drug is used in several immune-mediated inflammatory diseases (117, 118). This demonstrates that the functional outcome of TCR stimulation can be altered by manipulating the second signal without directly blocking the TCR itself.

### 7.3 Phosphatases as potential therapeutic targets

Another potential level of intervention is modulation of protein phosphatases that regulate TCR signaling. This approach, however, is substantially more complex than direct kinase inhibition.

Autoimmune pathology has been linked to dysregulation of PTPN22, PTPN6/SHP-1, PTPN11/SHP-2, PTPRC/CD45, PTPN2, PP2A, DUSP2, DUSP22, and other phosphatases (119). Their functions differ substantially: CD45 exerts both positive and negative effects, SHP-1 predominantly limits TCR signaling, whereas SHP-2 function depends on the context of its recruitment.

The term “phosphatase inhibitors” is therefore too broad when applied to autoimmune therapy. It is more appropriate to refer to selective pharmacological modulation of phosphatases. Depending on the molecule and cell type, therapeutic benefit may arise either from inhibiting pathologically increased phosphatase activity or from restoring or enhancing the function of a negative regulator.

Selective phosphatase modulation remains clinically less mature than co-stimulation blockade, but genetic associations between these regulators and autoimmune diseases support its therapeutic potential (119).

A particularly attractive strategy would be to target context-dependent phosphatase complexes that regulate TCR signaling in defined lymphocyte populations rather than the immune system globally. In principle, this could correct an abnormally altered activation threshold while reducing the risk of systemic immunosuppression.

### 7.4 From nonspecific immunosuppression to restoration of antigen-specific tolerance

A long-term goal of autoimmune therapy is restoration of antigen-specific tolerance through tolerogenic autoantigen presentation, enhancement of regulatory T-cell function, and generation of genetically engineered antigen-specific Treg cells.

The TCR-Treg concept is particularly promising: regulatory T cells are endowed with a defined TCR specificity. Unlike systemic immunosuppression, these cells could preferentially become activated in tissues where the relevant autoantigen is presented and locally suppress the pathological immune response (120).

The fundamental principles discussed above- TCR affinity, pMHC density, signal strength and duration, CD28 involvement, IL-2-dependent support of Treg cells, and cellular metabolic state-are directly relevant to the development of such cell products (120).

### 7.5 Immune checkpoint inhibitors as a means of controlling TCR signaling

In oncology, the therapeutic objective is fundamentally different. Tumor-specific T cells are often present in the patient and can recognize tumor pMHC, but their function is constrained by chronic antigenic stimulation and activation of inhibitory mechanisms. One of the most successful approaches has been blockade of the immune checkpoints CTLA-4 and PD-1/PD-L1 (121, 122).

Antibodies against CTLA-4 primarily relieve constraints on CD28-dependent co-stimulation, whereas PD-1/PD-L1 blockade acts predominantly on already activated tumor-reactive cells in peripheral tissues and the tumor microenvironment (121, 122).

Thus, immune checkpoint inhibitors do not change T-cell antigen specificity and do not directly increase the affinity of the TCR for pMHC. Their principal effect is to weaken inhibitory signaling and lower the functional threshold required for an existing TCR signal to be translated into a full T-cell response.

If a tumor lacks sufficient immunogenic pMHC, has lost HLA expression or antigen-processing capacity, or if the corresponding TCR clonotype is absent, relief of PD-1-dependent inhibition alone cannot generate an effective antitumor response.

### 7.6 Engineering TCR-like signal strength in CAR-T cells

One approach to deliberately tuning a TCR-like signal is to alter the number and functional configuration of ITAMs in synthetic receptors. Experimental variation of ITAM multiplicity has shown that increasing the number of functional motifs improves the efficiency with which receptor-ligand engagement is converted into a productive cellular response and may reduce the minimum number of receptor interactions required for activation. Conversely, reducing the number of functional ITAMs raises the activation threshold and can thereby increase selectivity for cells with high target-antigen density (44). This principle is directly relevant to CAR-T therapy because it allows antigen-density sensitivity to be altered without changing the specificity of the antigen-recognition domain itself.

In CAR-T cells, changing the number and position of active CD3ζ ITAMs can affect not only proximal signal magnitude but also cell fate. Calibration of ITAM signaling can shift the balance among effector differentiation, memory formation, and CAR-T cell persistence. In addition, altering the number of functional ITAMs can move the antigen-density threshold required for activation: stronger ITAM signaling favors recognition of targets with lower antigen expression, whereas reducing the number of active ITAMs raises this threshold (123, 124). Thus, the optimal strategy is not to maximize ITAM number or activity but to tune signal strength to the density of the target antigen, the required effector response, and the desired persistence of the cell product.

Physiological mechanisms of co-stimulation also provide direct engineering guidance. Because CD28 can enhance Lck recruitment and accelerate ZAP-70 activation within TCR microclusters (52), differences between CD28- and 4-1BB-containing CARs should be considered not only as differences in late metabolic programs but also as differences in the kinetics and amplitude of early signaling. This provides a route to tailoring the activation threshold to the expected density of the tumor antigen.

Another level of retuning concerns resistance to chronic signaling. Lynn et al. showed that c-Jun overexpression in CAR-T cells increases expansion potential, reduces terminal differentiation, and enhances antitumor activity in models of chronic tonic signaling (125). This example illustrates that cell therapy can be improved not only by altering the receptor itself but also by strengthening transcriptional programs that counteract the consequences of sustained signaling.

### 7.7 TCR-T cells as a direct clinical application of TCR biology

For the subject of this review, TCR-engineered T cells (TCR-T) represent the most direct translation of fundamental TCR research and form part of the broader class of engineered T-cell platforms under clinical development (87, 115, 126–128). T cells are genetically engineered to express a TCR with predefined specificity for a tumor peptide presented by a particular HLA molecule. Unlike CARs, TCR-T cells retain the natural TCR-CD3 complex and can recognize intracellular tumor proteins through their peptide-HLA complexes (126–128).

In the SPEARHEAD-1 trial, the MAGE-A4-specific product afamitresgene autoleucel (afami-cel) produced an objective response in 39% of patients with HLA-A*02-positive synovial sarcoma (129). In 2024, afami-cel (Tecelra) became the first FDA-approved genetically modified TCR-T cell therapy (130).

This milestone is important not only because it introduced a new therapeutic product, but because it provided clinical proof that the natural TCR-pMHC recognition mechanism can be exploited therapeutically in solid tumors (129, 130).

TCR-T efficacy depends on TCR-pMHC affinity and kinetics, TCR and HLA expression, pMHC density, antigen processing, coreceptor involvement, metabolic fitness, persistence, coinhibitory signaling, and the rate at which exhaustion develops (126–128). Consistent with this multifactorial view, antigen recognition by HER2/neu-specific TCR-T cells was associated with activation of T-cell signaling and proliferation programs, together with increased secretion of IL-2, granzyme B, TNF-α, and IFN-γ (131).

Current reviews of TCR-T therapy emphasize that efficient target recognition alone is insufficient for durable control of solid tumors; sustained antigen presentation, adequate T-cell infiltration, and resistance to the immunosuppressive microenvironment are also required (126–128).

### 7.8 T-cell engagers as external tuning of TCR/CD3 signaling

Principles of molecular TCR signal tuning apply not only to cellular products. Bispecific T-cell engagers create an artificial contact between CD3 on a T cell and a tumor target, thereby triggering TCR/CD3 signaling without requiring a clonotypic antigen-specific TCR. The efficiency of this synapse depends not only on the presence of the two targets but also on the affinity of the CD3-binding arm, avidity, valency, epitope geometry, tumor-antigen density, and the kinetics of formation and dissociation of the intercellular complex.

Experimental tuning of CD3 affinity has shown that reduced or intermediate CD3 affinity can preserve effective tumor-cell lysis while decreasing systemic cytokine release and improving *in vivo* biodistribution (132). Thus, the same functional-window principle applies to T-cell engagers: maximal binding strength does not necessarily provide the best therapeutic index.

A distinct class is represented by TCR-bispecific molecules such as ImmTACs, in which an affinity-optimized soluble TCR recognizes a tumor pHLA while an anti-CD3 fragment recruits an effector T cell. For such molecules, the balance between pHLA and CD3 affinities critically influences activation strength and specificity; combining a strong target-binding TCR with intermediate CD3 affinity may be preferable to maximizing both interactions simultaneously (133). These findings directly extend the concepts of kinetic proofreading and signal tuning from the physiological TCR to non-cellular T-cell-redirecting platforms.

Extending the bispecific principle to multispecific T-cell-engager formats makes it possible to combine recognition of a tumor target with simultaneous engagement of CD3 and a co-stimulatory receptor. For example, a CD19×CD3×CD28 trispecific antibody enhanced T-cell activation, accelerated tumor-cell lysis, and supported memory-associated differentiation features compared with a CD19×CD3 bispecific counterpart in preclinical models, illustrating that not only binding but also the quality of the second signal can be tuned (134).

Beyond affinity and valency, the geometry of the resulting immunological synapse is itself a critical parameter. Comparison of HER2×CD3 T-cell-engager formats showed that intermembrane distance and conformational flexibility independently influence signaling efficiency and cytotoxicity; closer contacts and optimal flexibility favor more productive synapse organization. Thus, epitope topology and the spatial architecture of the molecule are independent signal-tuning variables alongside CD3 affinity (135).

### 7.9 A functional signaling window rather than maximal affinity

Collectively, data from TCR-T and CAR-T cells support a common principle: a therapeutic construct should be evaluated according to the position and breadth of its functional signaling window rather than maximal affinity or maximal early-signal amplitude. In practice, this means relating antigen/pMHC density to proximal signaling, cytotoxicity, cytokine secretion, and resilience to repeated stimulation; detailed criteria for such evaluation are described above. For CARs, additional variables include ITAM number and the architecture of co-stimulation (44, 88, 92–100, 123).

### 7.10 Affinity heterogeneity and polyclonality as a prospective hypothesis for TCR-T therapy

The natural antigen-specific T-cell response is polyclonal and includes clonotypes with different TCR-pMHC interaction properties. Such affinity heterogeneity may be functionally important because binding strength determines not only the threshold for initial activation but also the kinetics of proliferation, differentiation, and exhaustion. Within a polyclonal response to a single tumor-associated antigen, cells with stronger TCR-pMHC interactions preferentially acquired proliferative phenotypes and progressed more rapidly toward terminal exhaustion, whereas weaker-binding clones more often retained a progenitor-like state; at the same time, stronger binding provided better early tumor control (99).

These observations suggest that the optimal TCR-T product may not necessarily consist of a single clonotype with the highest possible affinity, or even a single preselected “optimal” affinity, but rather a composition of T cells expressing receptors for the same target pMHC with different yet functionally acceptable affinity and kinetic characteristics. In principle, such a system could provide a functional division of labor: higher-affinity clonotypes may support a rapid effector response and recognition of cells with low pMHC density, whereas clonotypes with intermediate or lower binding strength may retain greater proliferative reserve, a higher activation threshold, and greater resistance to chronic antigenic stimulation. This is not simply a quantitative change in the “volume” of the same signal; differences in interaction duration, completeness of ITAM phosphorylation, and passage through kinetic proofreading steps can alter the relative contribution of individual TCR signaling branches.

Direct evidence that a mixed-affinity TCR-T product is superior remains limited, and this strategy should therefore be regarded as a prospective hypothesis rather than an established therapeutic principle. Its biological plausibility is nevertheless supported by related experimental observations. In 2026, Warmuth et al. showed in CAR-T cells recognizing the same epitope that combining high- and low-affinity receptors preserved the antitumor activity of the high-affinity fraction while reducing features of exhaustion and inflammatory toxicity; during chronic stimulation, low-affinity cells were more resilient and became increasingly represented in the mixed product over time (136). Pétremand et al. likewise showed that a combined product comprising several tumor-reactive TCR clonotypes achieved better tumor control than individual TCRs at a suboptimal dose, supporting the possibility of a therapeutic benefit from TCR polyclonality, although the clonotypes in that study were not deliberately selected to span an affinity gradient for a single pMHC (137).

A conceptual parallel can be drawn with the polyclonal humoral response, whose robustness is determined not by a single “best” antibody but by a collection of clones with different binding properties and epitope specificities. For TCR-T therapy, a potential advantage of an affinity-diverse product may lie not primarily in broader epitope coverage but in creating multiple functional modes of recognizing the same antigen. Testing this hypothesis will require direct comparison of mono- and polyclonal TCR-T products standardized for total cell dose, specificity for the same pMHC, affinity distribution, minimum detectable antigen density, repeated stimulation, AICD, exhaustion, and *in vivo* persistence.

### 7.11 Overcoming exhaustion and improving persistence of therapeutic T cells

The next stage of cell-therapy development concerns not so much further amplification of the initial signal as preservation of function after repeated tumor encounters. Practical strategies include modulation of checkpoint signaling, preservation of TCF1+ states, optimization of metabolic fitness, and reinforcement of AP-1-dependent programs; the latter is exemplified by c-Jun engineering, which increased CAR-T resistance to chronic stimulation (108, 111, 125).

Complete removal of negative regulators may be hazardous: these pathways constrain antitumor function but also protect cells from excessive activation and may support persistence under chronic stimulation. Retuning signaling thresholds is therefore preferable to eliminating negative regulation altogether (86, 102, 103, 105, 108).

In practical terms, this shifts the emphasis from eliminating negative regulators to preserving a functional signaling window during repeated stimulation, thereby maintaining cytotoxicity without accelerating progression to AICD or terminal dysfunction.

### 7.12 Personalized control of TCR signaling

In the future, TCR-oriented therapies will likely increasingly account for individual features of the receptor-antigen-cell system.

Relevant parameters for TCR-T therapy include HLA genotype, tumor antigens and mutations, peptide-HLA density, heterogeneity and processing of the target antigen, TCR properties, the starting T-cell phenotype, checkpoint status, and the metabolic tumor microenvironment.

Genome editing makes it possible to introduce a therapeutic TCR, remove the endogenous TCR, and modify inhibitory mechanisms. However, increasing the number of genetic interventions requires strict control of antigen discrimination and of the risks of off-target or on-target/off-tumor toxicity (128).

### 7.13 Outstanding questions and future directions

A key unresolved challenge is to establish a quantitative link between receptor biophysical parameters and clinically meaningful function. K_D_ and k_off_ values, pMHC density, ITAM number, Lck/CD45 levels, and co-stimulatory state are measured using different methods and in different experimental systems; no unified model yet reliably predicts from these parameters the response threshold, risk of AICD, exhaustion, and persistence of a particular cell product. This limits translation of fundamental TCR biology into standardized criteria for selecting therapeutic receptors.

A second barrier concerns antigen discrimination and safety. For TCR-T cells, critical variables include HLA restriction, interpatient variability in antigen processing and pMHC density, molecular mimicry, and cross-recognition; for CARs and T-cell engagers, analogous variables include surface-antigen density, synapse geometry, and the affinity of the CD3-binding component. Functional panels are needed that simultaneously assess sensitivity to low target density and reactivity toward closely related physiological antigens.

A third question is how to preserve durable function without excessive signal amplification. Engineering changes that increase initial cytotoxicity may simultaneously accelerate AICD, terminal differentiation, or epigenetic fixation of exhaustion. A promising direction is combined tuning of receptor architecture, ITAM number, co-stimulatory modules, and transcriptional programs of resilience, followed by testing in repeated-stimulation models rather than only in single-round cytotoxicity assays.

Finally, signaling assessment in clinically relevant contexts remains insufficiently standardized. The same TCR or CAR can produce different outcomes in naive, central-memory, effector, or previously exhausted cells and at different levels of CD4/CD8, CD28, Lck, CD45, and inhibitory receptors. Next-generation T-cell products will require integrated assays that combine receptor biophysics, quantitative pMHC/antigen burden, proximal phosphosignaling, repeated periodic stimulation, and metabolic fitness. Such systems should enable a shift from empirical construct selection to predictable engineering control of TCR signaling.

## 8 Conclusion

TCR signaling is not a linear cascade but a flexible, multiparametric system of cellular decision-making. It integrates the spatial organization of TCR-CD3, pMHC-binding kinetics, Lck and ZAP-70 activity, dynamic regulation by CD45 and SHP phosphatases, co-stimulatory and inhibitory receptors, metabolic programs, and the duration of antigenic signaling (29–31, 99).

Fundamental TCR research has already yielded several classes of clinically relevant strategies for controlling T-cell signaling. CD80/CD86-CD28 blockade modifies the activation threshold in autoimmune disease (117, 118), checkpoint therapy reduces coinhibition (121, 122), whereas CAR-T, TCR-T, and T-cell-engager platforms reconstruct or redirect TCR/CD3 signaling in different ways (126–130, 132–135, 138–140).

The next stage of T-cell therapy is likely to depend not on simply increasing receptor signal strength but on quantitatively and spatially tuning it. An optimal system should combine sensitivity to low target-antigen density with preserved specificity, controlled proximal-signal amplitude, resilience to repeated stimulation, and sufficient proliferative reserve (44, 52, 88, 92–100, 123, 125, 132, 133).

The central translational concept is therefore a shift from the strategy of “activating or suppressing the T cell” toward tuning the functional range of TCR signaling- its threshold, duration, and context.
